# CLINICAL outcomes and loss to follow-up among people living with HIV participating in the *NAMWEZA* intervention in Dar es Salaam, Tanzania: a prospective cohort study

**DOI:** 10.1186/s12981-017-0145-z

**Published:** 2017-03-28

**Authors:** Hellen N. Siril, Sylvia F. Kaaya, Mary Kay Smith Fawzi, Expeditho Mtisi, Magreat Somba, Japheth Kilewo, Ferdinand Mugusi, Anna Minja, Anna Kaale, Jim Todd

**Affiliations:** 1 Department of Psychiatry and Mental Health, Muhimbili University of Health and Allied Sciences, Dar es Salaam, Tanzania; 2 Management and Development for Health (MDH), Plot number 802, Mwai Kibaki Road, 255 Dar es Salaam, Tanzania; 3 Department of Global Health and Social Medicine, Harvard Medical School, Boston, MA USA; 4 African Academy for Public Health (AAPH), Dar es Salaam, Tanzania; 5 Department of Epidemiology, Muhimbili University of Health and Allied Sciences, Dar es Salaam, Tanzania; 6 Department of Internal Medicine, Muhimbili University of Health and Allied Sciences, Dar es Salaam, Tanzania; 7 National Institute for Medical Research (NIMR), Mwanza, Tanzania; 8 Department of Epidemiology and Biostatistics, Kilimanjaro Christian Medical University College, Moshi, Tanzania; 9 Population Health Department, London School of Hygiene and Tropical Medicine, London, UK

**Keywords:** Psychosocial, NAMWEZA, HIV/AIDS, ART, LTFU, PLH, Dar es Salaam, Tanzania

## Abstract

**Background:**

Psychosocial factors have been linked with loss to follow-up (LTFU) and clinical outcomes among people living with HIV (PLH), however little is known about the effect of psychosocial support on LTFU among PLH in treatment and care. The purpose of this study was to explore the effect of *NAMWEZA* (“Yes, together we can”) friends’ psychosocial support intervention on clinical outcomes and LTFU among PLH. *NAMWEZA* is based on a novel program using “appreciative inquiry”, positive psychology approaches to empower, promote positive attitudes and foster hope.

**Methods:**

PLH participating in the *NAMWEZA* intervention in HIV care clinics in Dar es Salaam Tanzania were compared with non-exposed PLH obtained from facilities that routinely collect clinical information and both followed longitudinally for 24 months. Baseline sociodemographic, clinical measures (CD4 cell count, hemoglobin (HGB), weight), and LTFU measures were collected. Chi square, Fisher’s exact tests, and t-tests were used to compare the frequencies for categorical variables and the means of continuous variables from the intervention and the comparison groups to identify variables that were significantly different across the two groups. Random effects models were performed to examine the bivariate associations between the intervention status and clinical outcomes.

**Results:**

At the end of 24 months of follow-up mean CD4 count and HGB levels increased significantly in both intervention and comparison groups (p = 0.009 and p < 0.0001, respectively). Weight increased significantly only in the intervention group (p = 0.003). Cumulative LTFU was three times higher in the comparison compared to the intervention (p < 0.001) group. Having a low CD4 count, extremes of weight, low HGB, younger age, and male gender were significantly associated with LTFU among the unexposed group, while being on ART for duration of 12 months or more was protective against LTFU in those intervened.

**Conclusion:**

Among PLH on ART, exposed or not exposed to *NAMWEZA* intervention, clinical care outcomes improved over time. LTFU was much higher in the comparison group with factors commonly known to predict LTFU only apparent in the comparison group. *NAMWEZA* could be a promising peer-facilitated model to reduce LTFU among PLH in care that can be integrated in ART services; however, more research is needed to evaluate its longer term effects.

## Background

Although there has been increased public and patients’ awareness of the benefits of antiretroviral therapy (ART) globally [1], in many countries attrition from ART care programs has been high, with loss to follow-up (LTFU) of patients listed as a leading cause [2–6]. However, ART can only be effective, resulting in virological suppression, if PLHs are highly adherent (commonly described as taking equal to or over 95% of ART medication) [7]. This level of drug adherence is very difficult to attain for PLH who are unable to sustain scheduled clinic follow-up visits, as studies indicate that the majority of patients with LTFU stop their ART. Stopping ART has been reported to quickly increase the risk of developing high serum viral load and other adverse health consequences [2, 5, 8, 9] including ART drug resistance, progression of HIV to AIDS, premature mortality, and re-infection, or infection of others with resistant strains [2–4, 10, 11].

In sub-Saharan Africa, LTFU is a factor reported to account for attrition from most ART programs than other causes [12–14] and varies for different groups of PLH. For instance LTFU ranges from 19 to 89% among women accessing Prevention of Mother-To-Child HIV transmission (PMTCT) services and 22% among HIV infected children [15]. In Tanzania, where this study was conducted, LTFU among PLH enrolled in ART care programs is estimated to be 49% and it varies with duration in ART care. About 18 and 36% of PLH in Tanzania are reported to be LTFU at the end of their first and third year respectively after starting ART. A report published by the Ministry of Health and Social Welfare in 2012 indicated that attrition of patients due to death accounted for only a small percent of PLH that was lost from ART treatment (5–8%). Most attrition was due to other unknown causes resulting in the majority of the LTFU [14, 16].

Some of the factors reported to contribute to LTFU from ART care in Tanzania include individual factors such as advanced clinical and immunological disease stage, younger age, malnutrition, low education, depression, and poor psychological support [17–21]. The World Health Organization (WHO) reported other individual risk factors for LTFU from ART care commonly reported from resource-limited countries, including feeling better, pill burden, treatment fatigue, work as well as home responsibilities, and migration—mobile populations (‘silent transfers’)—to other ART service providers [22]. Likewise system/infrastructural factors contributing to LTFU from ART care have been reported from East Africa. These include longer turnaround time of laboratory tests, drug stock outs, and clinics operating for less than 4 days a week [23] LTFU therefore results from complex individual and programmatic/system challenges facing PLH and ART programs that require greater effort to adapt effective interventions to maximize the benefit of ART programs for PLH and the role of ART as an HIV transmission prevention approach [3, 20, 24, 25].

Although a number of studies have reported LTFU and other ART treatment outcomes among PLH in Tanzania, most did not include assessments of additional interventions provided to PLH apart from routine ART care. Thus there is still limited information on effective interventions for PLH in care and receiving ART for reducing LTFU and improving other HIV treatment outcomes [8, 14]. In particular, those that influence psychological factors that have been reported to predict HIV risk behaviors, poor adherence, low retention and poor treatment outcomes among PLH [26]. Providing psychological counselling, offering psychoeducation, facilitating psychosocial support and providing support to PLH to reduce psychosocial challenges including depression, anxiety, relationship/family turmoil and poor communication have been reported to increase adherence to ART [21, 27–30]; however, not much has been reported on how psychosocial interventions may impact LTFU and other HIV treatment outcomes in Tanzania.

The primary objective of our study was to examine associations between exposure to the *NAMWEZA* intervention and HIV clinical outcomes as well as LTFU. In addition, factors associated with LTFU were assessed among PLH in Dar es Salaam, Tanzania.

## Methods

### Study design

We conducted a prospective cohort study involving 416 HIV infected adult participants exposed to the *NAMWEZA* intervention and 408 HIV-positive unexposed ‘comparison group’. The data for this analysis was collected from January 2012 to December 2013 and were obtained from NAMWEZA intervention electronic records and National HIV care electronic database known as *CTC2* available at each of participating sites.

### Eligibility criteria

Both *NAMWEZA* exposed and unexposed participants were eligible if HIV infected, 18 years of age and above, living in the catchment area of the HIV care and treatment center (CTC), registered for HIV care and treatment at the study CTCs, initiated on ART and had used ART for at least 3 months (enrolment criteria for both NAMWEZA exposed and controls), and resident in the Kinondoni District. The ART regimen and other standard HIV related health care services were provided similarly between the two groups. Exclusion from the study occurred if poor health prohibited participant’s recruitment, if they had not started ART and for the NAMWEZA exposed, if unable to provide informed consent for participation, and not planning to remain resident for at least 2 years in the Kinondoni District.

### Methods of follow up

Both the intervention and control participants were seen monthly at the clinic for routine ART care during the time period of follow-up (January 2012 to December 2013). HIV care included adherence counseling, seeing a physician for any health concerns, providing laboratory tests as scheduled and any other tests that a physician might feel the patient needs at that visit. Each clinic has trained and experienced community based health workers (CHWs) who tracked missing PLH. At the time of the study, data were collected prospectively from the clinical databases. The CHW worked with the clinic data clerks under the supervision of the clinic nurse in-charge to obtain daily lists of PLH who miss their scheduled clinic visits. After obtaining the list, they followed up the missing patients through phone calls (1 week after missing a scheduled clinic visit) and a home visit 2 weeks after a missed clinic appointment if phone calls were unsuccessful and or the patient didn’t come to the clinic. The CHW provided reports that were entered into the CTC2 database daily to update patients’ vital status (i.e. not ascertained, ascertained alive or died). The CHW continued to track those not found for three or more months after which if vital status could not be ascertained they are declared LTFU.

### Exposure variable

*NAMWEZA,* pronounced as two linked words *NAM*-*WEZA* (Yes I can!), is an intervention that includes 10 once weekly structured sessions aimed at empowering PLH to become HIV prevention change agents in their communities. Psychosocial issues related to relationships and living with HIV as well as change agent’s communication skills building occurred using an Appreciative Inquiry (AI) approach. The AI model engages stakeholders in self-determined change and was first used to change organizational/system behavior. It involves searching for the best in people, and the world around and systematic discovery of what gives a system ‘life’ when it is most effective and capable in economic, ecological, and human terms. Participants receive training sessions that targets developing the art and practice of asking questions that strengthen an individual or a system’s capacity to heighten positive potential [31, 32]. The AI model components used in the process of delivery of *NAMWEZA* group sessions, included strategies that built participants skills to ask reflective and appreciative questions about what is working well in themselves, people’s lives specifically their social network members and through the answers, identify and affirm abilities in self and others; and using dreams about the future and backlighting to focus action plans for change in the here and now. Participants used appreciative inquiry to explore their abilities potentials, dream for and draw feasible action plans for the future including abilities to access and manage microfinancing keep healthy relationships, Burgan for safer sexual practices, and handling HIV infection and disclosure. Also *NAMWEZA* fostered resilience and social support through building participant’s communication skills in the use of “I” rather than “You” statements to improve clarity and reduce conflict when communicating with others. All sessions include repeated exercises and role plays practiced during the sessions, with each session building on the previous one. Participants were encouraged to practice the skills of each session at home and give feedback before the beginning of the next session. This approach aims to increase self-confidence and self-esteem as a way of exploring, highlighting, and developing skills as resources within each session theme [33]. The thematic content of *NAMWEZA* included themes that ranged from exploring love, relationships, sex, and feelings in session 1 to action planning in a closing session 10 (see Table 1).

**Table 1:** Summary of the contents of each NAMWEZA Training Session

Session 1	An introductory session: which include welcoming the participants, setting ground rules participants sharing life stories and group lived values, the story of the 5th province, whom do you hear calling your name and an introduction of appreciative inquiry in communication, how to spot abilities of others, dreaming positively for future, and how to be wise
Session 2	Titled Love relationship and emotions: include more training to use appreciative inquiry in communication, exploring values of love and how to view emotions from self and others (anger, sadness), as an invitation and a communication instead of an attack, and how to communicate back
Session 3	Titled Valuing others and self: include sharing experiences that influence life, how we value ourselves, how we value our own words and imagined negotiation
Session 4	Titled Happiness, safer sexual relationships, healthy living; include drawing a map of safer and happy living, appreciative inquiry, following direction education and discussions on HIV transmission and prevention myths and facts about HIV, following directions, positive networking and condom use
Session 5	Titled Developing assertiveness skills; revisions and role plays on appreciative inquiry, ability spotting, exploring hopes and fear, taking control during communications by using proper posture, eye contact and voice tones, I see, I feel, I want, putting things into practice drawing bodies as roadmap for sexually learning
Session 6	Titled Deepening and expanding assertiveness skills: More revision and role plays on appreciative inquiry, ability spotting, wider assertive skills building including manipulative how to manage an ambiguous situation, pushing the line during communication and entrepreneurial future
Session 7	Titled Disclosure 1; of an HIV positive status; confidentiality, appreciative inquiry, good care giver, human rights, telling and listening, how to become a good listener
Session 8	Titled Disclosure 2; challenges and positive possibilities, taking care of self, how to tell family and close relatives, sexual partners, supporting each other and spiders web
Session 9	Titled Visualizing; targets on focusing the participants to explore, reflect skills and resources in them and others, and create a microeconomic plan. This involve visualizing and drawing hoped for community/work, Appreciative inquiry exploring income generating skills including how to access and manage microfinances
Session 10	Titled The future; This is the final session that focuses on what has been learned during the all the other sessions by revisiting the tree of life and future possibilities for advocacy. Participants revisit and add on the previously tree of live adding their abilities and building on dreams and creating action plans for the future, mental gifts, tree of abilities and how to move in the community

Sessions were highly participatory and active learning occurred with the opportunity to practice learned skills and provide feedback in subsequent sessions. Training occurred in age and gender specific groups (women <35 years of age; men ≤35 years; women ≥35 years; and men ≥35) to foster group cohesion through shared experiences. Groups were facilitated by trained peer PLH. *NAMWEZA* exposed participants attended 1–10 sessions, with an average of 7.7 sessions; while the comparison group did not attend any sessions. All the sessions were available to all participants, who were informed of the dates and times and expected to attend all the 10 sessions. In addition, both the *NAMWEZA* exposed PLH and the comparison group received routine ART care including monthly prescriptions for medications, as well as other aspects of clinical care and ART adherence counseling sessions.

### Outcome variables

Loss to follow-up (LTFU), immunological (CD4), and other clinical outcomes including weight, hemoglobin level, and number of scheduled clinic visits attended were examined. For this study a patient was LTFU if he/she did not attend three scheduled clinic visits in three consecutive months with no reported/documented death or transfer out [34].

### Data collection

Outcome data were obtained from the CTC electronic database which contains routinely collected clinical data for PLH accessing care at each of the participating sites. Data for sociodemographic measures, duration on ART, and all outcome variables including CD4, weight, and hemoglobin levels; as well LTFU measures were extracted from the database at 8, 16, and 24 months of follow-up.

### Data analysis

Descriptive statistics for sociodemographic factors and clinical variables were estimated at baseline, including frequencies for categorical data as well as means and standard deviations for continuous variables. Chi square, Fisher’s exact tests, and t-tests were used to compare the frequencies for categorical variables and the means of continuous variables from the intervention and the comparison groups to identify variables that were significantly different from the two groups. Random effects models were performed to examine the bivariate associations between the intervention status and clinical outcomes, including CD4 cell count, weight change, HGB levels, and LTFU. In the multivariate models variables that had a p value of 0.2 or less at baseline for the intervention and comparison groups were controlled for. In addition, a missing variable indicator was included to address missing data and logistic regression models were used to predict factors associated with LTFU for intervention and comparison groups. A Kaplan–Meier survival curve was used to estimate retention among the intervention and comparison groups. We also examined factors associated with LTFU using Cox proportional hazards regression models. SAS statistical software (version 9.3) was used for the analysis [35].

## Results

All 450 HIV-positive participants of *NAMWEZA* intervention were eligible to participate. Thirty-four (7.5%) of the exposed participants had absent or incomplete clinical information and were dropped from the analysis. The final number of participants in the intervention group included in this study was 416 (92%). A total of 408 PLH were identified from the CTC2 database based on the same eligibility criteria.

At baseline, only gender and age variables were statistically significant different from the intervention and comparison groups (p = 0.03 and <0.0001, respectively). The other demographic characteristics were not significantly different (Table 2). Seventy-two percent and 77% of the participants from intervention and comparison groups respectively were females. The mean age was 46 and 37 year for intervention and comparison groups, respectively. About half (49%) of intervention and slightly above 50% (52%) of the comparison group, reported to be living with a sexual partner in a marital or cohabiting relationship. Majority of the participants in both the intervention and comparison groups reported that they were self-employed; 38 and 41% respectively, followed by those who were employed (35 and 36%) respectively, a few were house wives/husbands (9 and 11%) respectively and unemployed were 15% in the intervention and 16% in the comparison group (Tables 2, 3).

**Table 2:** Baseline sociodemographic characteristics for PLH receiving HIV treatment in Dar es Salaam, Tanzania

Characteristic	Comparison group, N = 408 n (%)/mean (SD)	Intervention, N = 416 n (%)/mean (SD)	p value
District of residence			
Kinondoni	387 (95.3)	412 (99.8)	
Ilala	10 (2.5)	1 (0.24)	0.0897^1^
Temeke	9 (2.2)	0 (0.0)	
Sex			
Male	92 (23.0)	116 (28.0)	0.0301^2^
Female	316 (77.0)	300 (72.0)	
Mean age	37 (9.1)	46 (9.7)	<0.0001^2^
Age groups in years			<0.0001^2^
<30	30 (11.3)	30 (7.2)	
≥30	236 (88.7)	386 (92.8)	
Marital status^a^			
Lives with partner (married or cohabiting)	208 (52.0)	205 (49.2)	0.3000^2^
Single (never married or widowed)	192 (48.0)	203 (48.7)	
Employment status			
Employed	142 (34.8)	148 (35.6)	
Self-employed	166 (40.7)	156 (37.5)	0.6510^2^
House wife/house husband	35 (8.6)	45 (10.8)	
Unemployed	65 (15.9)	67 (16.1)	

^1^p value obtained from Fisher’s exact test

^2^p value was obtained from Chi Square test

^a^A total of eight participants from the comparison group and eight from the intervention with missing data were omitted

**Table 3:** Baseline clinical characteristics for PLH receiving ART care in Dar es Salaam, Tanzania

Characteristics	Comparison	Intervention	p value^1^
Mean CD4 (SD)	270 (173.4)	308 (184.1)	0.76
Immune suppression			
Severe immunosuppression (CD4 < 200)	116 (28.5)	96 (23.1)	0.09
Moderate immune suppression (CD4 = 200 to <350)	119 (29.2)	138 (33.2)	
Not immune suppressed (CD4 ≥ 350)	173 (42.4)	182 (43.8)	
Mean hemoglobin (SD)	10 (2.1)	9 (1.9)	0.09
Low hemoglobin(anemia) mean (SD)	11 (2.6)	5 (1.2)	
Severe anemia (<8.5)	60 (14.7)	52 (12.5)	0.03
Moderate anemia (8.6 to <10)	179 (43.9)	278 (88.8)	
Mild anemia (10 to <12)	122 (29.9)	81 (19.5)	
No anemia (>12)	44 (9.0)	91 (22.0)	
Mean weight (kg) (SD)	60 (11.9)	61 (12.7)	0.33
Weight groups (kg)			0.21
<45	72 (18.6)	21 (5.1)	
45–60	88 (21.6)	50 (12.0)	
>60	227 (55.6	341 (82.0)	
Mean duration on ART at baseline, mean (SD)	18 (17.0)	20 (16.0)	0.23

N-intervention group = 412 and 367 control group: A total of 21 participants in the comparison group and 4 in the intervention had missing data and were omitted

^1^p-value was obtained from Chi Square test

For the clinical outcome variables baseline CD4 cell count in the intervention and control groups was 308 and 270 cells/mm^3^, respectively; and mean hemoglobin (HGB) levels were 9 g/dl for the intervention and 10 g/dl for the comparison group. The mean body weight of the participants was 61 and 60 kg in the intervention and control groups respectively. Similar to most demographic variables, the clinical parameters were not significantly different in the two groups (Table 3).

The mean duration on ART medication was 20 months in the intervention and 18 months in the comparison group (p = 0.23). The median follow up time was 20.2 months in the intervention and 23.3 months in the intervention group (Table 6). The average number of routine clinic visit made was significantly higher at 22.8 visits in the intervention group, compared to 16.9 visits in the comparison group (p = 0.003) (Table 6).

**Fig. 1:**
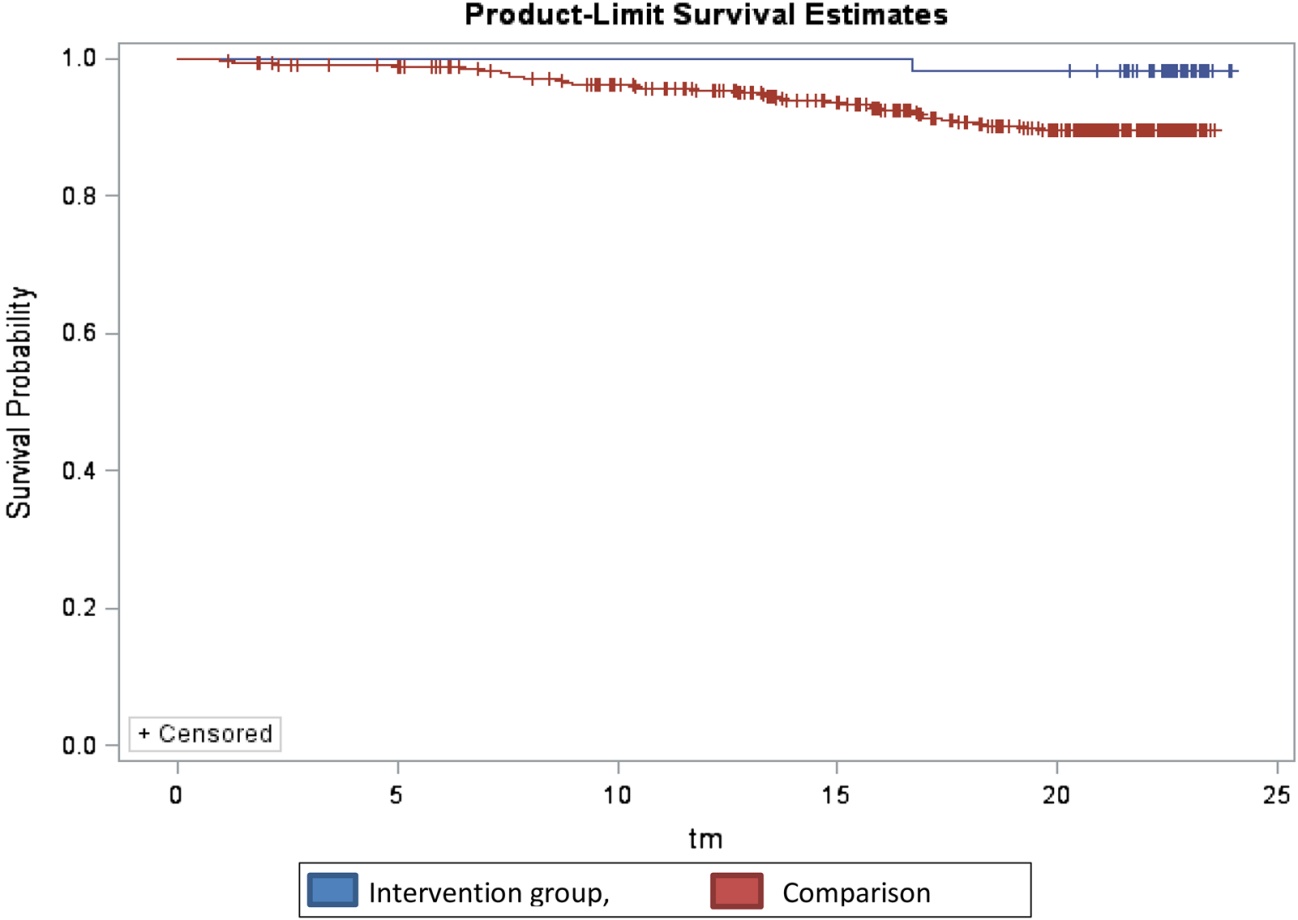
Kaplan Meier curve; showing LTFU in the intervention group (*blue*) and Comparison group (*red*)

Multivariate analyses showed that CD4 counts increased significantly in both the intervention and comparison groups over time (p = 0.0024 and 0.047, respectively). However, women in both groups had significantly faster rates of CD4 count increase of about 41 cells/mm^3^ per month compared to men (p = 0.005) (Table 4). Over time, participants in the intervention group had significantly higher weight gain of (0.270 kg per month compared to 0.140 kg in the comparison group (p = 0.003). At the end of follow-up, the proportion of PLH who were LTFU was significantly lower in intervention (4.8%) compared to (14.7%) in the control group (p = 0.002) (Table 6). More PLH were retained in the intervention compared to the control group as depicted by the Kaplan–Meier curve in Fig. 1. In the intervention group, the proportion of LTFU was significantly higher (57%) among the participants who attended 1–6 *NAMWEZA* training sessions than those who attended 7–10 sessions (43%), p = 0.033 (Table 6).

**Table 4:** Univariate and multivariate analyses of intervention vs. comparison group

Covariate of interest	Univariate		Multivariate	
	Effect estimate	p value	Effect estimate	p value^1^
CD4 count (cells/mm^3^)				
Intervention vs. comparison CD4 change over time	24.6 (−37.02, 86.23)	0.4337	26.9 (−36.31, 90.19)	0.4036
Intervention	2.9 (1.19, 4.64)	0.0009	2.7 (0.96, 4.45)	0.0024
Comparison group	2.8 (0.84, 4.75)	0.0051	2.8 (0.87, 4.79)	0.047
Age			−0.58 (−1.72, 0.56)	0.3196
Gender			40.56 (12.41, 68.72)	0.0048
Weight (kg)				
Intervention vs. comparison Weight change over time	0.9 (−6.97, 8.71)	0.8270	−2.64 (−9.91, 4.620)	0.4757
Intervention	0.14 (−0.073, 0.35)	0.2000	0.27 (0.09, 0.44)	0.0025
Comparison group	0.15 (−0.03, 0.31)	0.0968	0.14 (−0.34, 0.31)	0.1234
Age			0.08 (−0.01, 0.17)	0.0681
Gender			−0.79 (−3.04, 1.46)	0.4892
Hemoglobin (HGB) (in g/dl)				
Intervention vs. comparison HGB change over time	0.12 (−1.60, 2.05)	0.8109	0.23 (−1.61, 2.06)	0.7268
Intervention	0.03 (−0.02, 0.09)	0.2726	0.031 (−0.02, 0.09)	0.0429
Comparison group	0.02 (−0.01, 0.05)	0.1154	0.020 (−0.01, 0.05)	0.0437
Age (in years)			0.01 (−0.01, 0.03)	0.2026
Gender			−0.61 (−1.05, −0.18)	0.006
Lost to follow-up				
Intervention vs. comparison	0.62 (0.51, 0.76)	<0.0001	0.64 (0.52, 0.78)	<0.001
LTFU change over time				
Intervention	0.92 (0.91, 0.93)	<0.0001	1.01 (1.01, 1.03)	0.0033
Comparison group	0.93 (0.51, 0.76)	<0.0001	0.92 (0.92, 0.93)	<0.001
Age			0.99 (1.00, 1.001)	0.1737
Gender			0.95 (0.89, 1.02)	0.3169

^1^p value obtained from random effects model

For LTFU, five out of the six factors that were associated with LTFU, were only significant in the control participants, but not the intervention group participants. These included: low CD4; PLH with severe immune suppression (CD4 < 200 cells/mm^3^ had higher risk of LTFU (p < 0.0001); extremes in weight, where those with extreme low or high weights (<45 and >60 kg) were at higher risk of LTFU (p = 0.02); low hemoglobin; participants with lower hemoglobin levels (<10 mg/dl) showed higher risk of LTFU (p = 0.01). Male gender was associated with higher risk of LTFU (p = 0.04) and younger age (<40 years) was associated with higher LTFU risks (p = 0.03). In the intervention group, PLH with longer duration of ART use, experienced less LTFU (p < 0.0001), while in the comparison group, persons with longer duration on ART were at higher risk of LTFU (p < 0.0001) (see Tables 5, 6).

**Table 5:** Factors associated with LTFU among the intervention and comparison group participants

Factor	Intervention				Comparison group			
	Univariate HR (95% CI)	p value	Multivariate HR (95% CI)	p value	Univariate HR (95% CI)	p value	Multivariate HR (95% CI)	p value^1^
CD4		0.01		0.12		<0.001		<0.0001
<200	0.75 (0.54, 0.03)		1.55 (1.11, 2.15)		3.20 (2.52, 4.53)		2.13 (1.63, 2.75)	
200 to <350	0.31 (0.21, 0.45)		0.86 (0.58, 1.27)		2.52 (1.98, 3.21)		1.83 (1.41, 2.36)	
350+	Reference		Reference		Reference		Reference	
Weight WT		0.83		0.65		0.001		0.0200
<45	0.98 (0.99, 1.02)		0.29 (0.02, 0.32)		0.96 (0.68, 1.36)		1.48 (0.95, 2.31)	
45–60	Reference		Reference		Reference		Reference	
60+	0.94 (0.97, 2.03)		0.34 (0.03, 2.01)		1.60 (1.31, 1.96)		2.01 (1.63, 2.49)	
HBG		0.74		0.09		<0.001		<0.000
<8.5	1.11 (0.60, 2.07)		1.93 (0.91, 3.67)		0.40 (0.38, 0.43)		3.24 (2.97, 3.55)	1
8.5 to <10	0.68 (0.47, 0.99)		0.10 (0.06,0.16)		0.44 (0.42, 0.46)		0.48 (0.46, 0.51)	
10 to <12	1.91 (1.32, 2.77)		1.17 (1.11, 0.26)		0.57 (0.55, 0.60)		1.00 (0.95, 1.05)	
12+	Reference		Reference		Reference		Reference	
ART duration		<0.0001		<0.0001		<0.0001		<0.001
<12	Reference		Reference		Reference		Reference	
12+	0.39 (0.27–0.57)		0.42 (0.28–0.63)		2.34 (1.98–0.21)		2.51 (1.84–0.43)	
Gender						<0.000		0.0400
Male	0.02 (0.01–10.47)	0.85			1.49 (1.22–0.82)	1	1.28 (1.01–0.63)	
Female	Reference				Reference			
Age		0.89				0.002		0.0300
<30	3.31 (0.01–0.67)				0.87 (0.64–0.99)		0.73 (0.54–0.99)	
30 to <40	3.23 (0.01–0.23)				0.92 (0.79–0.15)		0.89 (0.69–0.14)	
40 to <50	Reference				Reference		Reference	
50+	5.21 (0.02–0.23)				0.24 (0.06–0.38)		0.14 (0.06–0.35)	

^1^p value was obtained from Chi square test

**Table 6:** Cumulative attendance of scheduled clinic visits, LTFU, LTFU by sessions at and follow up time the end of the 24 months among the intervention and comparison groups

	Control	Intervention	p value^1^
Average number of scheduled visits in months, n (SD)	16.9 (6.27)	22.8 (2.01)	0.0030
Cumulative lost to follow up by end of 24 months, n (%)	60 (14.7)	20 (4.8)	0.0016
LTFU among those who attended 1–6 sessions, n (%)		12 (60)	0.0330
LTFU among PLH attending 7–10 sessions, n (%)		8 (40)	
Median follow up time (months)	23.3	20.2	0.6800

^1^p value obtained by Chi square test

## Discussion

This study examined associations between exposure to a peer facilitated psychosocial intervention and clinical outcomes among PLH receiving ART. Participants exposed to the *NAMWEZA* intervention saw significant improvement in clinical outcomes that were not seen among those who did not receive the *NAMWEZA* intervention. Weight gain, CD4 counts, and hemoglobin all significantly increased among *NAMWEZA* participants, whereas smaller non-significant gains were seen in the comparison group. The *NAMWEZA* intervention had lower LTFU rates and participants were more likely to remain in treatment. The *NAMWEZA* protective effects against LTFU had a dose–response effect with lower rates observed among those who attended more of the *NAMWEZA* sessions.

In studies that have reported ART clinical outcomes among PLH, low CD4, younger age, male gender, low HGB, and weight were commonly reported to be associated with LTFU [8, 14, 24, 36]. In this study these risk factors for LTFU were all independently significantly associated with LTFU among PLH in the comparison group but were not found for intervention group participants. These findings are similar to other studies reporting ART treatment outcomes and rates of LTFU [8, 14, 36]. In addition, the cumulative rates of LTFU were three times higher in the comparison group as compared to the intervention group. This is also reflected in the average number of clinic visits which was significantly lower among the comparison group.

This variation could be related to the effect of exposure to the psychosocial intervention (*NAMWEZA*) in addition to routine ART care, and suggests that *NAMWEZA* may have had an effect on reducing LTFU in HIV care. Such interventions could be considered for integration within the ART care and treatment clinics to reduce LTFU.

The observed increase in CD4 counts and hemoglobin levels in both groups was expected since participants in both groups were receiving ART medications and were likely to show clinical improvements related to the duration of treatment [15, 37, 38]. However, the intervention group participants showed a higher increase in both CD4 counts and hemoglobin levels compared to the control group. Close follow up of PLH during the *NAMWEZA* intervention could have prompted more clinic visits and greater adherence to ART by exposed participants compared to unexposed group participants. Furthermore, the intervention addressed psychosocial concerns which have been reported to hinder adherence to ART and retention in care [21]. This is similar to what was observed in Zimbabwe and Zambia where a psychosocial intervention known as “mothers2mothers” (m2m) was found to improve retention and adherence to the Option B+ antiretroviral regimens among HIV infected women [39, 40]. Although these studies were based on pregnant and breastfeeding women, the participants were taking a full regimen of ART similar to participants in this study. Better management of psychosocial concerns in the intervention group could also explain the significantly higher weight gain over time for *NAMWEZA* when comparing participants to those in the unexposed group.

To our knowledge this is the first intervention study where PLH receiving ART medication were provided a psychosocial intervention that showed improvements in selected ART treatment outcomes. The study took place in an area which has a high HIV prevalence, especially among women, and a significant number of PLH receiving ART medications for varying durations.

There are a number of limitations for this study. The routinely collected clinical data used to select participants for this study had many gaps in information since the purpose of data was for clinical care and not research. We excluded from the analysis of this study (both intervention and control arms), participants who had more than 50% missing information in the 12 months before the study, perhaps limiting the representativeness of the study results. Although the study is observational, major confounders have been controlled for in the study, and selection of participants according to the same eligibility criteria may have reduced potential bias.

## Conclusions

The findings suggest that providing additional psychosocial support to PLH receiving ART can reduce LTFU. Such support needs to be formalized and integrated with HIV treatment and care. In addition, working with peer supporters in this context was feasible and is likely generalizable to other resource-limited settings with a significant burden of HIV. Facilitating such interventions could in the long run reduce expenditures due to adverse health outcomes resulting from LTFU in HIV care.
